# Modeling risk of *Sclerotinia sclerotiorum*-induced disease development on canola and dry bean using machine learning algorithms

**DOI:** 10.1038/s41598-021-04743-1

**Published:** 2022-01-17

**Authors:** F. Shahoveisi, M. Riahi Manesh, L. E. del Río Mendoza

**Affiliations:** 1 Department of Plant Pathology, North Dakota State University, Fargo, ND 58108 USA; 2 School of Engineering, Campbell University, Buies Creek, NC 27506 USA

**Keywords:** Biotic, Environmental impact

## Abstract

Diseases caused by the fungus *Sclerotinia sclerotiorum* are managed mainly through fungicide applications in canola and dry bean. Accurate estimation of the risk of disease development on these crops could help farmers make spraying decisions. Five machine learning (ML) models were evaluated in classification and regression modes for predicting disease establishment under different air temperatures and leaf wetness duration conditions. Model algorithms were trained and tested using 20-fold cross validation. Correspondence between predicted and observed values were measured using Cohen’s Kappa (classification) and Lin’s concordance coefficients (regression). The artificial neural network (ANN) algorithms had average accuracies ≥ 89% (classification) and R^2^ ≥ 88% (regression) on canola and dry bean and their correspondence agreements were ≥ 0.83, which is considered substantial to almost perfect. In contrast, logistic regression algorithms had accuracies of 88% for dry bean and 78% for canola; other models were similarly inconsistent. Implementation of ANN models in disease warning systems could help farmers with spraying decisions. At the same time, these models provide insights on temperature and leaf wetness requirements for development of *S. sclerotiorum* diseases in these crops. Results of this study show the potential of ML models as tools for epidemiological studies on other pathosystems.

## Introduction

Sclerotinia stem rot of canola (*Brassica napus*) and white mold of dry beans (*Phaseolus vulgaris*) are caused by *Sclerotinia sclerotiorum*. In addition to these, *S. sclerotiorum* affects more than 500 plant species including several economically valuable crops such as soybean, sunflower, lettuce, and sugar beet^1,2^. The disease cycle tightly follows the life cycle of this pathogen. Resting structures, called sclerotia, germinate carpogenically to produce apothecia in which ascospores will be formed. Upon release into the air, ascospores that sediment on flowers germinate and infect the petals^3^. After pollination, the pathogen in casted infected-petals that land on plant tissues may colonize them causing water-soaked and soft lesions. As lesions develop on leaves, they darken and expand into the branches and the main stem. Infected stem portions turn bleached and the epidermis shreds. When lesions girdle the stem, the plants wilt, and die. At the end of the season or under unfavorable environmental conditions, survival structures called sclerotia are formed in or on infected portions of the stem. Sclerotia overwinter on soil or stubble where they can start new infections in the next season^4^. While epidemics caused by this pathogen on dry bean, canola, and most other plant species are established by ascospores^5^ as described, in some crops, mycelia emerging from sclerotia also could infect plant roots causing wilt of infected plants.

Epidemiological models in plant pathology are developed to characterize the role environmental variables play on aspects of the life cycle of plant pathogens or on the development of the diseases they cause. For *S. sclerotiorum-*induced diseases, some of these variables are temperature, leaf wetness duration, and soil moisture^6^. These models improve our understanding of the disease and can be used to develop better disease management tools. Several models have been developed to describe disease development in time or space and it has been customary for researchers to fit multiple models to the same data set to identify the one that provides the best fit^7–11^. A different situation occurs when the risk of disease development is modeled using a single tool, e.g., logistic regression, which has been by far the most popular tool to develop these predictive models in the last 20 years^12–16^. While logistic regression is considered a machine learning (ML) classification technique; other ML techniques only have been sporadically used for plant disease modeling or not used at all.

ML techniques may contribute to substantial advances in development of epidemiological models that estimate risk of plant disease development^17^. ML techniques can be classified in three groups, supervised, unsupervised, and reinforcement. In the supervised learning group, which is the most used, researchers indicate the patterns that should be looked for, e.g., environmental conditions that result in disease incidences above 20%. Examples of techniques in this group are linear regression (LNR) and logistic regression (LGR), linear discriminant analysis (LDA), support-vector machine (SVM), classification regression (CLR) and decision tree (DT), artificial neural network (ANN), naïve Bayes classifier (NBC), and k-nearest neighbor (KNN). The latter three techniques also could be used as unsupervised learning techniques. In the unsupervised learning group, the techniques simply classify data in clusters that share similar characteristics; examples of this group include K-means clustering, hierarchical clustering, anomaly detection, principal component analysis, independent component analysis, and a priori algorithms. In the third group, techniques are model-free or model-based, and the algorithms learn by trial and error. Some of these techniques have less restrictions than others, e.g., no basic assumptions, and have become more accessible thanks to advances in computing; however, the accuracy of the models they produce may still be influenced by the uniqueness of the data sets used to develop them, as well as due to experimental error gathered during data collection and analysis. A direct comparison of the accuracy of these models should be made only when the models are developed using the same data set^18^.

In a recent review, Yang and Guo^19^, highlighted the application of ML techniques in discovery of plant disease resistance genes as well as in plant disease detection and indicated that few reports had been made on their use for evaluation of disease development onset. A short literature review identified a few of the papers that have used ML techniques to assess disease onset. The techniques reported include ANN, DT, random forest (RF), and SVM^20–25^. In ANN, which is a technique commonly used in forecasting systems and data classification^26^, information from independent variables is entered as input layer. The effect of each variable on the dependent variable is “weighted” in one or more hidden layers. An excitatory response is considered a positive weight and an inhibitory response is considered a negative weight^27^. These weights are summed and then an activation function reigns the output to be usually between 0 and 1. The DT technique consist of nodes that test the value of certain attributes or features, terminal nodes or leaf nodes that correspond to predicted outcomes, and branches or links that connect input nodes to the next nodes or to the leaves. In the initial step, a tree is generated for the full dataset and then every leaf is processed separately. During processing, data are recursively split using Gini Index as metric and Iterative Dichotomiser 3 which applies entropy function and information gain as metrics^27–29^. RF models use bootstrap or bagging aggregation methods to reduce variation in the prediction model. In this model, several decision trees are constructed from multiple bootstrapped samples of the training data. The prediction result from each decision tree is subjected to voting and the most voted prediction class is selected^30^. The SVM model measures the similarity between the data used for training and the new dataset. Different similarity kernel functions can be used in SVM algorithm such as linear, polynomial, quadratic, radial basis function, and sigmoid^31^.

To the best of our knowledge, there is no report on the application of ML algorithms on prediction of diseases caused by *S. sclerotiorum* using environmental factors. Therefore, experiments were designed (i) to compare common ML techniques, like ANN, RF, DT, LGR, and SVM, for their ability to predict Sclerotinia stem rot/white mold disease incidence on canola and dry bean using regression analyses; and (ii) to identify the most accurate ML algorithm using classification analyses.

## Results

### Disease incidence

The variances of canola and dry bean trials were homogenous (*P* = 0.8348 and 0.7251, respectively) and therefore, a combined analysis within each crop was conducted. The analysis of variance for canola (Table 1) indicated the interaction between wetness duration and incubation temperature was not significant (*P* = 0.1768) but the main effects of both factors were significant (*P* < 0.0001). The optimum incubation temperature was 25 °C with an average incidence of 88%. Reducing the incubation temperature to 15 or 20 °C lowered the incidence by almost 20% while increasing it to 30 °C reduced incidence by 43% (Table 2). The analysis of variance for dry bean data (Table 1) indicated the interaction between the evaluated wet and dry periods was not significant (*P* = 0.2738) but the main effects of both factors were significant (*P* < 0.0001). Incidence increased significantly (*α* = 0.05) when the length of the wet incubation period increased to 16 h (Table 2) but increasing it from 8 to 12 h did not result in significant increases. Similarly, extending the dry incubation period from 18 to 24 h resulted in an approximately 30% reduction in incidence but extending it from 12 to 18 did not affect incidence (Table 2). Providing constant wet conditions, without dry periods, led to the second highest disease incidence at 78%.

**Table 1:** Analysis of variance of the effect of incubation temperature and interrupted leaf wetness period and of interrupted leaf wetness on diseases incidence caused by *Sclerotinia sclerotiorum* ascosporic infections on canola and dry bean, respectively.

Crop	Sources of variation	Degrees of freedom		F-value	*P*-value
		Numerator	Denominator		
Canola	Temperature	4	95	38.16	< 0.0001
	Leaf wetness	3	95	11.01	< 0.0001
	Temperature × leaf wetness	12	95	1.41	0.1768
Dry bean	Leaf wetness	2	128	13.64	< 0.0001
	Dry period	2	128	13.07	< 0.0001
	Leaf wetness × dry period	4	128	1.30	0.2738

Analysis was conducted using the GLIMMIX procedure of SAS (version 9.4). The studies were conducted for 10 and 8 days, respectively.

**Table 2:** Main effects of discontinuous leaf wetness duration and incubation temperatures on incidence (%) of foliar lesions caused by *Sclerotinia sclerotiorum* ascosporic infection on canola and dry bean plants.

	Canola		Dry bean	
Factors	Levels	Incidence (%)	Levels	Incidence (%)
Incubation temperature (°C)	10	28 d	–	–
	15	73 b	–	–
	20	66 b	–	–
	25	88 a	–	–
	30	50 c	–	–
Leaf wetness (hours/cycle)	6	51 c	8	56 b
	10	53 bc	12	54 b
	14	65 ab	16	78 a
	18	75 a		
Leaf dryness (hours/cycle)	–	–	12	74 a
	–	–	18	67 a
	–	–	24	46 b

On canola, a successive wet and dry period adds to a cycle of 24 h; in dry bean, the cycle does not necessarily add to 24 h. Incidence values are least square means that represent 24 and 34 observations on canola and dry bean plants, respectively. Incidence was measured after 8 and 10 days of incubation of canola and dry bean plants, respectively.

Incidence means followed by same letters in a factor are not statistically different (*α* = 0.05) from each other according to the Tukey–Kramer test.

A “–” indicates levels of the factor were not tested.

### Classification analyses

**Figure 1:**
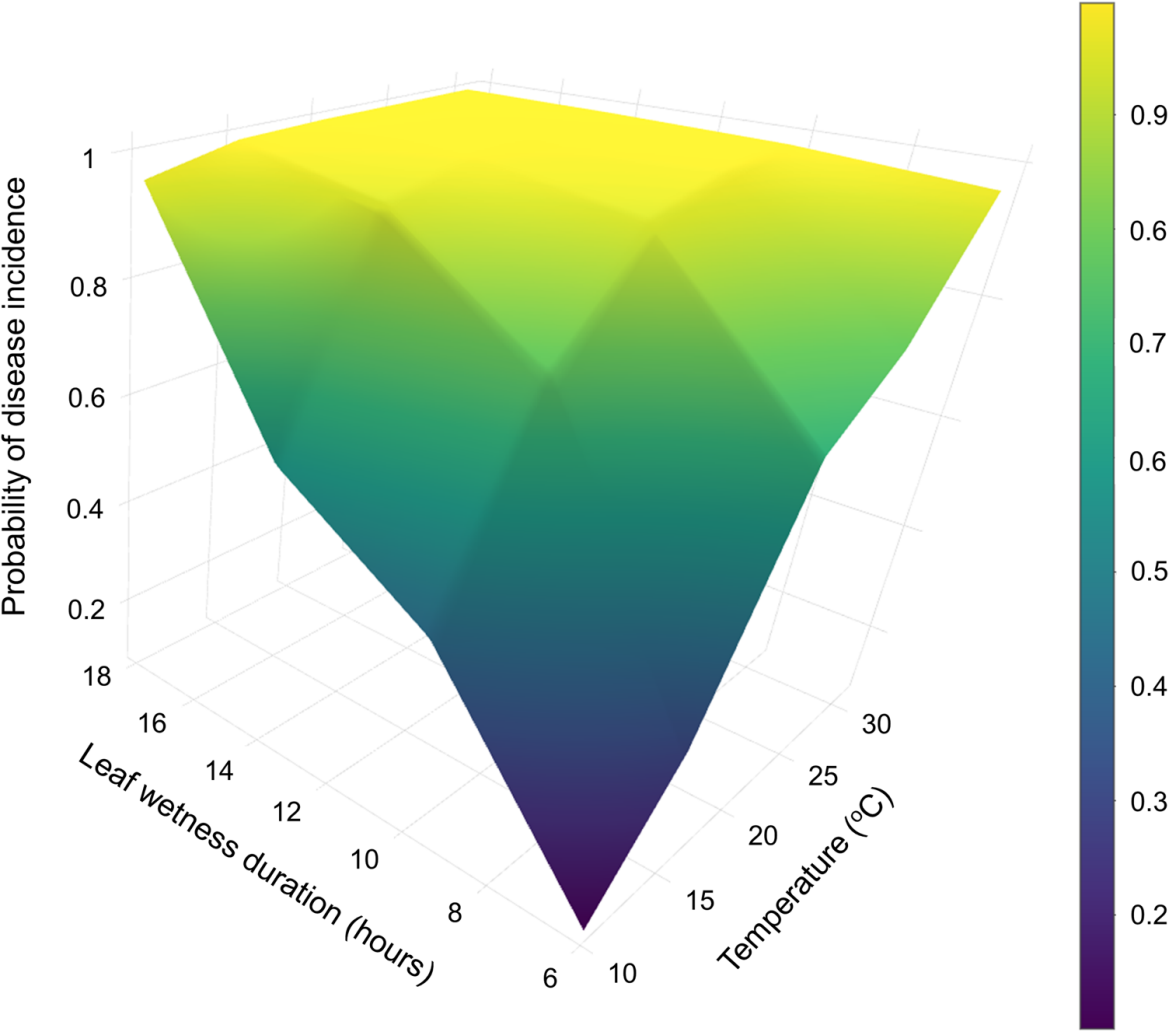
Prediction probabilities of Sclerotinia stem rot development on canola using classification artificial neural network (ANN). Temperature, leaf wetness duration, and total time from the inoculation were used as predictors of the model. Figure shows the probabilities estimated nine days after inoculation.

For canola, the ANN and SVM models showed the highest accuracy and precision followed by RF (Table 3, Supplementary Table S1). When compared to LGR, the ANN and SVM models were 11 to 10% more accurate and 12 to 11% more precise, respectively. However, all three models had similar recall percentage which ranged between 91 and 92%. The ANN and SVM models had F-scores of 91% while LGR had a score of 85%. The RF model had greater accuracy, precision, and F-score than LGR but similar recall values. Figure 1 shows the probabilities of disease development predicted by ANN using temperature, leaf wetness, and total time from the inoculation as predictors.

**Table 3:** Evaluation of fitness of artificial neural networks (ANN), support-vector machine (SVM), random forest (RF), decision trees (DT), and logistic regression (LGR) machine-learning models used in classification analyses of canola and dry bean data sets that associated incubation temperature and duration of leaf wetness conditions with incidence of Sclerotinia stem rot disease.

Study	Models	Model fitness metrics				
		Accuracy (%)	Precision (%)	Recall (%)	F-score (%)	AUC (%)
Canola	ANN	89	91	92	91	93
	SVM	88	90	92	91	91
	RF	86	88	91	89	89
	DT	78	83	84	83	72
	LGR	78	79	91	85	86
Dry bean	ANN	92	90	93	91	95
	SVM	90	87	93	90	96
	RF	85	85	82	83	94
	DT	83	82	82	82	82
	LGR	88	86	89	87	95

AUC represents the area under the receiver operating characteristic curve.

For dry bean, ANN performed consistently better than LGR by showing greater accuracy, precision, recall and F-score (Table 3, Supplementary Table S1). The SVM model was slightly superior to LGR; however, in contrast to the canola data, LGR was superior to the RF model. The ANN model had accuracy, precision, recall, and F-scores ranging between 90 and 93%, while LGR values ranged between 86 and 89%. Detailed results of the predictions during model development are presented in Supplementary Table S2. The ANN model showed substantial to almost perfect agreement^32^ between predicted and observed events with average Kappa coefficients of 0.75 and 0.83, for canola and dry bean, respectively, whereas the coefficients for LGR ranged between moderate (0.5) and almost perfect (0.83). The SVM model showed substantial agreement (0.73–0.80) for both crops while DT and RF had the lowest coefficients (Table 5).

### Regression analyses

In the canola study, the ANN algorithm was superior to all other models with higher R^2^, and smaller root mean square error (RMSE) and mean absolute error (MAE) values (Table 4). RF was identified as the second-best model followed by DT. The LNR and SVM models provided a significantly lower fit to the data, explaining 31 to 35% of the variation in disease incidence. Visual association between actual and predicted incidence values are represented on Table S3. ANN also was superior to all models in the dry bean data set with R^2^ of 95% and smaller RMSE and MAE values (Table 4, Supplementary Table S3). The LNR and SVM models fit the dry bean data much better than the canola data, but both were still outperformed by the DT and RF models. When model predictions were compared to actual outcomes, ANN was clearly superior to all other models with a moderate to substantial agreement between observed and predicted values^33^ as indicated by average Lin’s concordance coefficient of 0.94, 0.98 for canola and dry bean, respectively. The second-best models were RF (*ccc* of 0.87 and 0.95) and DT (*ccc* of 0.86 and 0.94) for canola and dry bean, respectively. LNR and SVM had lower coefficients (Table 5). Detailed results of the predictions during model development are presented in Supplementary Table S3.

**Table 4:** Statistical fitness metrics of artificial neural networks (ANN), support-vector machine (SVM), random forest (RF), decision trees (DT), and linear regression (LNR) machine-learning models used in regression analyses of canola and dry bean data sets that associated incubation temperatures and duration of leaf wetness conditions to incidence of Sclerotinia stem rot disease.

Study	Models	R^2^ (%)	Root mean square error	Mean absolute error
Canola	ANN	88	7.84	6.09
	RF	77	10.91	8.20
	DT	73	11.91	8.19
	LNR	35	18.43	14.52
	SVM	31	18.97	13.91
Dry bean	ANN	95	5.82	4.36
	RF	90	8.46	5.52
	DT	88	9.54	6.80
	LNR	74	13.70	11.48
	SVM	70	14.90	12.34

**Table 5:** Concordance coefficients for classification (Kappa) and regression (Lin’s *ccc*) models for correspondence between observed and predicted outcomes of artificial neural networks (ANN), support-vector machine (SVM), random forest (RF), decision trees (DT), logistic regression (LGR), and linear regression (LNR) machine-learning models used to characterize the effect of leaf wetness and incubation temperature on incidence of Sclerotinia stem rot of canola and dry bean.

Study	Models	Kappa	*ccc*
Canola	ANN	0.75	0.94
	RF	0.68	0.87
	DT	0.51	0.86
	LGR	0.50	–
	LNR	–	0.53
	SVM	0.73	0.49
Dry bean	ANN	0.83	0.98
	RF	0.70	0.95
	DT	0.67	0.94
	LGR	0.83	–
	LNR	–	0.86
	SVM	0.80	0.80

## Discussion

As pointed by Skelsey^34^, application of ML in agriculture has been overwhelmingly oriented towards recognition of images, whether it is of weeds, fruits, flowers, or of plant diseases^35–38^, with very few applications being made on the estimation of the risk of disease development. One of the first publications on the latter area was made by Kaundal^22^ who compared the conventional multiple regression to the generalized regression neural networks, and SVM to predict rice blast. In their work, Kaundal et al.^22^ observed that SVM produced a more accurate algorithm than the other methods. To the best of our knowledge, this is the first time that five ML models are compared for their efficacy to predict development of Sclerotinia-induced diseases on canola and dry beans.

The flexibility and versatility of ML models were in evidence in the study reported in this manuscript. ANN models were more efficient than LGR when used either in classification or regression modes and in general provided substantial to almost perfect levels of correspondence between observed and predicted events^32,33^. LGR is considered the tool of choice for development of predictive plant disease risk models^12–16^. The superiority of ANN over LGR was highlighted earlier by Dreiseitl and Ohno-Machado^39^ who indicated that in 36 of 72 instances models developed using ANN outperformed those from LGR, while LGR models were superior to ANN models only in five instances. A similar result was observed by Paul and Munkvold^24^, who used it to model the association between gray leaf spot severity using multiple environmental and cultural factors. Further, Chakraborty et al.^20^ reported the application of ANN in modeling the relationship between severity of anthracnose of *Stylosanthes scabra* and several weather variables; their results showed that the best ANN model had the accuracy of > 85%. In the present study, the fitness and accuracy of two other ML models, SVM and RF, were a close second in classification and regression mode analyses, respectively. SVM produced superior models than LGR for the canola set although the LGR models were better than SVM on the dry bean set. Using classification data, SVM produced models with 88% and 90% accuracy in canola and dry bean, respectively, while RF produced models with 85–86% accuracy in both data sets. The high performance of SVM and RF algorithms also have been reported by other researchers; for example, Mehra et al.^23^ reported an RF algorithm with accuracies of 93% and R^2^ of 79% that modeled *Stagonospora nodorum* blotch of winter wheat, while Wen et al.^25^ reported another for soybean rust that explained 76 to 87% of the total variation in spore movement.

Environmental factors such as temperature, relative humidity, precipitation, wetness duration, and wind speed have a determinant role on development of plant diseases and those caused by *S. sclerotiorum* are no exception*.* In this study, the impact of interrupted leaf wetness was explored. The range of temperatures evaluated in this study could not be considered extreme since incidences at 10 °C and 30 °C ranged between 28 and 50%. It is likely that the lack of significant interactions between leaf wetness and incubation temperature or length of the dry period is due to the resiliency of the pathogen to desiccation once it is in plant tissues^40^. Multiple prediction models for Sclerotinia diseases have been developed using weather components; for example, Mila et al.^15^ studied the role of air temperature and precipitation on the probability of SSR disease prevalence on soybean using LGR models. In their study, they used monthly means for both variables obtained from 320 weather stations distributed on four states and produced models with high explanatory powers that were like the ones reported for LGR models in our study. However, the predictive accuracy of ANN models was 11% and 4%, greater than the LGR models for canola and dry bean, respectively. Harikrishnan and del Río^14,40^ conducted studies on white mold disease of dry bean under growth chamber and field conditions and produced models with high explanatory power that ranged between 65 and 91% accuracy. Clarkson et al.^41^ studied the association between air temperature, relative humidity, and ascospore density on disease development on lettuce. The role of wind, air temperature and relative humidity on development of white mold on soybean was investigated by Willbur et al.^42^, who produced models with 81.8 to 87.9% accuracy. These tools can assist farmers with spraying decisions. To make these models available to farmers, they could be incorporated into disease-warning systems like the Sporecaster which became available to Michigan soybean growers in 2018^43^ or the Sclerotinia risk map that is available to North Dakota farmers^44,45^. The ANN model presented in this study accurately predicted disease development using environmental factors, e.g., wetness duration and/or temperature, and did it consistently in classification and regression modes, and for two different crops, canola, and dry bean. This flexibility is an indication that ML techniques could be used to model other stages of the disease progress such as apothecial development, spore dispersal, and infection process. The models developed for each stage could then be merged to generate a comprehensive forecasting system. However, model validation prior to full implementation should be conducted under different environmental conditions and locations to ensure its reliability.

In summary, results of this study highlight the potential of ML methods for the development of models that evaluate risk of plant disease development. ANN could predict disease development with high accuracy in classification and regression analyses on both crops, whereas the accuracies of other models, including LGR, were affected by the crop, the type of analysis, and the predictors used. Nevertheless, other ML techniques, e.g., RF, have produced excellent models in other pathosystems^21,23^. Thus, it could be said that more modern ML techniques albeit more complex than LGR, may be described as the “next generation” tools for modeling the risk of plant disease development.

## Materials and methods

### Data collection

Seed samples from the canola and dry bean cultivars used in this study were obtained from the respective NDSU breeding programs. Westar is an open-pollinated canola cultivar that was released in 1987 in Canada^46^. Westar is no longer available for commercial production but is routinely used as susceptible control in *S. sclerotiorum* trials in our program. ‘Maverick’ is a dry bean cultivar released by the NDSU dry bean breeding program in 1997^47^. Maverick is still commercially available and because of its susceptibility to *S. sclerotiorum*, it also is used as susceptible control in our trials. Since these materials were/are commercially available, voucher specimens were not deposited in publicly available herbaria. Use of these cultivars for research purposes is neither restricted nor regulated in any form by relevant institutional, national, and international guidelines and legislation.

#### Canola

Detailed process of inoculation and data collection is described by Shahoveisi and del Río Mendoza^16^. Briefly, canola flowers were collected from Sclerotinia stem rot-susceptible cultivar, Westar. Flowers were inoculated with dry ascospores of *S. sclerotiorum* isolate WM031 by placing them in the upper two layers of an Andersen spore sampler^48^ and then activating the sampler above groups of mature lab-produced apothecia. A total of ten inoculated flowers were placed on leaves of a canola plant per replication. Inoculated plants were incubated in different combinations of alternating wet and dry conditions in a period of 24 h (i.e., 6/18, 10/14, 14/10, and 18/6 of wet/dry hours). Experimental units were arranged using a randomized complete block design with three replications and the entire study was conducted twice. Plants were placed in closed plastic bags to maintain the leaf wetness for the required period and then opened to allow drying of the plants. At the end of the dry incubation period, plants were sprayed with distilled water and the plastic bags were closed. Disease incidence, expressed as the percentage of inoculated flowers that formed leaf lesions, was recorded at the end of each wet cycle starting 24 h after plant inoculation until 10 days post inoculation.

#### Dry bean

Dry bean flowers, cv. Maverick, were collected and inoculated with isolate WM030. The same inoculation method described for canola was used in this experiment. Five flowers were placed on the primary leaves of a dry bean seedling at the V-1 stage. These plants were subjected to alternating wet and dry incubation conditions but a wet and dry period did not necessarily add to a 24 h cycle (i.e., 8/12, 8/18, 8/24, 12/12, 12/18, 12/24, 16/12, 16/18, 16/24, and 24/0 of wet/dry hours). Each treatment was replicated four to five times in a completely randomized design with a single plant per replication. The study was conducted four times. Moist chambers set at 20 °C were used for wet incubation periods. Plants were transferred into a room set at 18 °C with relative humidity at 30 ± 5% at the end of each wet period. Starting the wet condition, leaves were sprayed with water until runoff. Disease incidence was recorded each time that plants were returned to moist conditions until 8 days post inoculation.

### Data analyses

#### Disease incidence

Maximum disease incidences, measured at the end of each study on both crops, were used to estimate the mean incidence for each treatment. Since the study conducted on dry bean plants was not a full factorial, the continuous wetness treatment was not included in the analysis of variance for incidence. The canola study was a full factorial. Homogeneity of variances of trials in each study was tested using Levene’s test at *α* = 0.05. Then combined analyses of variances were conducted using the GLIMMIX procedure of SAS software (version 9.4; SAS Institute, Cary, NC) where treatments were considered fixed effects and trials, replications, and their interactions with treatments were considered random effects. Tukey–Kramer mean separation test at *α* = 0.05 was conducted to compare the least square means of treatments.

#### Classification analyses

All statistical analyses were conducted using Orange software suite (version 3.24.0; University of Ljubljana, Slovenia). Supplementary Figure S1 represents the summary of the workflow system used by Orange 3.24.0 software for classification and regression analyses. For the canola study, temperature, wetness duration, and accumulated wetness time from inoculation were considered independent variables. These predictors were selected because the interaction of interrupted wetness duration and incubation temperature has been studied to a lesser extent^16^. Average disease incidences recorded over the period of the study across all trials and replications for each treatment were considered as the dependent variable (N = 131). For the dry bean study, wetness duration, dry period, and accumulated time were used as the independent variable and average disease incidence was the response variable (N = 60). Five supervised-learning classification models, ANN, RF, DT, SVM, and LGR were evaluated for their ability to model disease development. The training and testing of the models were conducted using 20-fold cross validation. Binomial datasets of the dependent variable were generated where incidence values less than or equal 20% were labeled as 0 to indicate no disease development while incidence values greater than 20% were labeled as 1 to account for disease development. This threshold was selected because incidences below this level do not reduce yields significantly^14^.

For the ANN model, one hidden layer with ten and five neurons and hyperbolic tangent, tanh, activation function, that adds non-linear property to the function, were used on canola and dry bean data sets, respectively. The numbers for maximum iterations and learning rate (α) were set at 2000 and 0.5 for canola and 2000 and 0.7 for dry bean, respectively. For the DT model, no limits were set to the tree depth and to the minimum number of instances in leaves (pruning). The node splitting was stopped after a 95% majority threshold was reached. For the RF model, 5 and 16 trees for canola and dry bean datasets were used, respectively. For the SVM model, radial basis function (RBF) kernel with numerical tolerance of 0.0001 and unlimited iteration were used for both crops. Minimum misclassification rate was obtained when the coefficient for the loss function (C) was set at 110 and 0.8 for canola and dry bean, respectively (this difference was due to sample size and data characterization). The epsilon (ε) value of 1 was used for canola while this threshold was 0.9 for dry bean dataset. LGR uses a logistic function to classify the data into binary values. Using one to several predictors, probabilities of an event, such as disease development, is calculated by this algorithm^16,49^. Two parameters of LGR algorithms are regularization function and cost strength that is the inverse of regularization parameter (λ). In both studies, regularization function was obtained using Ridge regression (L2) with cost strength of 5 and 50 for canola and dry bean, respectively. Table 6 summarizes the type of analysis and parameter estimates used for development of each model.

**Table 6:** Parameter estimates of artificial neural networks (ANN), decision trees (DT), random forest (RF), support-vector machine (SVM), logistic regression (LGR), and linear regression (LNR) machine-learning models used in classification and regression analyses.

Study/analyses	Models				
	ANN	DT	RF	SVM	LGR/LNR
Canola/classification	Hidden layers = 1	Pruning = none	Number of trees = 5	Loss function = 110.0, ε = 1.0	Regularization = ridge (L2)
	Neurons = 10	Node splitting = 95%	Replicable training = yes	Kernel = RBF, exp(− auto|x–y|^2^)	Cost strength = 5
	Activation function = tanh	Tree depth = unlimited	Tree depth = unlimited	Numerical tolerance = 0.0001	
	α (learning rate) = 0.5		Max number of considered features = unlimited	Iteration = unlimited	
	Max iteration = 100				
Dry bean/classification	Hidden layers = 1	Pruning = none	Number of trees = 16	Loss function = 0.8, ε = 0.9	Regularization = ridge (L2)
	Neurons = 5	Node splitting = 95%	Replicable training = yes	Kernel = RBF, exp(− auto|x–y|^2^)	Cost strength = 50
	Activation function = tanh	Tree depth = unlimited	Tree depth = unlimited	Numerical tolerance = 0.0001	
	α (learning rate) = 0.7		Max number of considered features = unlimited	Iteration = unlimited	
	Max iteration = 100				
Canola/regression	Hidden layers = 1	Pruning = none	Number of trees = 10	Loss function = 1.0, ε = 0.8	α (regularization parameter) = 1
	Neurons = 200	Node splitting = 95%	Replicable training = yes	Kernel = Linear	
	Activation function = tanh	Tree depth = unlimited	Tree depth = unlimited	Numerical tolerance = 0.0001	
	α (learning rate) = 0.7		Max number of considered features = unlimited	Iteration = unlimited	
	Max iteration = 2000				
Dry bean/regression	Hidden layers = 2	Pruning = none	Number of trees = 10	Loss function = 1.0, ε = 0.8	α (regularization parameter) = 1
	Neurons = 20	Node splitting = 95%	Replicable training = yes	Kernel = linear	
	Activation function = logistic	Tree depth = unlimited	Tree depth = unlimited	Numerical tolerance = 0.0001	
	α (learning rate) = 1		Max number of considered features = unlimited	Iteration = unlimited	
	Max iteration = 2000				

LGR was used in classification and LNR in regression analyses.

#### Regression analysis

Fitness of ANN, RF, DT, SVM, and LNR algorithms to the canola and dry bean datasets was evaluated. In both studies, average disease incidence across all trials and replications were used as the outcome. The training and testing were conducted using 20-fold cross validation. The performance of the algorithms was compared using their R^2^, RMSE, and MAE. R^2^ represents the percentage of the variation in the dependent variable that is explained by the predictors, RMSE indicates the model’s average error in prediction of the response variable for an observation, and similarly MAE corresponds to the prediction error of the model.

##### ANN

In the canola analysis, a hidden layer with 200 neurons were used and the activation function was set to tanh. Also, the maximum iterations were set to 2000 and the learning rate to 1. Learning rates closer to 1 result in a more radical weight modification. For dry bean, model parameters were as following: two hidden layers each with 20 neurons, logistic activation function, learning rate of 1, and maximum iteration value of 2000 (Table 6).

##### DT

Similar to the classification analysis, in both canola and dry bean regression studies, data splitting continued until the majority threshold reached 95% level. In addition, we held no limits on the maximal tree depth as well as the minimum number of splits in leaves.

##### RF

For canola and dry bean studies, 10 trees were used; and maximal number of considered features and maximal tree depth were set as “unlimited” (Table 6).

##### SVM

This algorithm was run using linear kernel function, with numerical tolerance set to 0.0001, and unlimited iterations. Minimum misclassification rate was obtained when penalty rate parameter was set at 1. A threshold of 0.90 was used for ε parameter in both studies (Table 6).

##### LNR

This model is the simplest ML algorithm that describes the linear relationship between the input (independent variable) and the output (dependent variable)^50^. In disease forecasting studies, multiple LNR models allow analyzing the regression between multiple predictors and the response variable. In both canola and dry bean analyses, the regularization parameter (α) was set at 1 (Table 6).

#### Model comparisons

To estimate the overall fitness of each model, confusion matrices of the models were obtained and metrics including area under the receiver characteristic curve (AUC), classification accuracy (CA), precision, recall, and F1 score were calculated. AUC indicates the ability of the model to distinguish the classes. A higher AUC represents a more accurate model in separating 0 and 1 classes. CA is the ratio of correct predictions (true positive and true negative) to the total number of predictions. Precision is calculated by dividing “true positive” by “total predicted positive” whereas recall is ratio of “true positive” to “total actual positive”. The recall proportion reflects the percentage of instances where the model correctly identified a case [true positive/(true positive + false negative)]. The F-score provides a harmonic mean between precision and recall and in a way, describes the overall accuracy of a model. The F1 score is calculated by the following formula:

$$F1=2 \times \frac{(Precision\times Recall)}{(Precision+Recall)}.$$

When expressed as percentage, the closest the precision, accuracy, recall, F1 score, or AUC, is to 100, the better the model is. To further visualize the relationship between actual and predicted incidences from each model, plots were produced and added as Supplemental Materials.

#### Model validation

To evaluate the predictive ability of each model, a bootstrapping procedure with replacement was implemented. The levels of correspondence between predicted and actual values for each model were estimated using the Kappa statistic^51^ for classification data and Lin’s concordance correlation coefficient^52^ for regression models. The Lin’s ccc and Kappa statistic were calculated for each crop separately.

## Supplementary Information

Supplementary Figure S1.

Supplementary Table S1.

Supplementary Table S2.

Supplementary Table S3.
