# Supplementary material for: Modeling risk of Sclerotinia sclerotiorum-induced disease development on canola and dry bean using machine learning algorithms
Source: Sci Rep. 2022 Jan 17;12:864. doi: 10.1038/s41598-021-04743-1 (PMC8764076; doi:10.1038/s41598-021-04743-1)
Supplement: Supplementary file 1 — Supplementary Figure S1. [file 41598_2021_4743_MOESM1_ESM.docx]

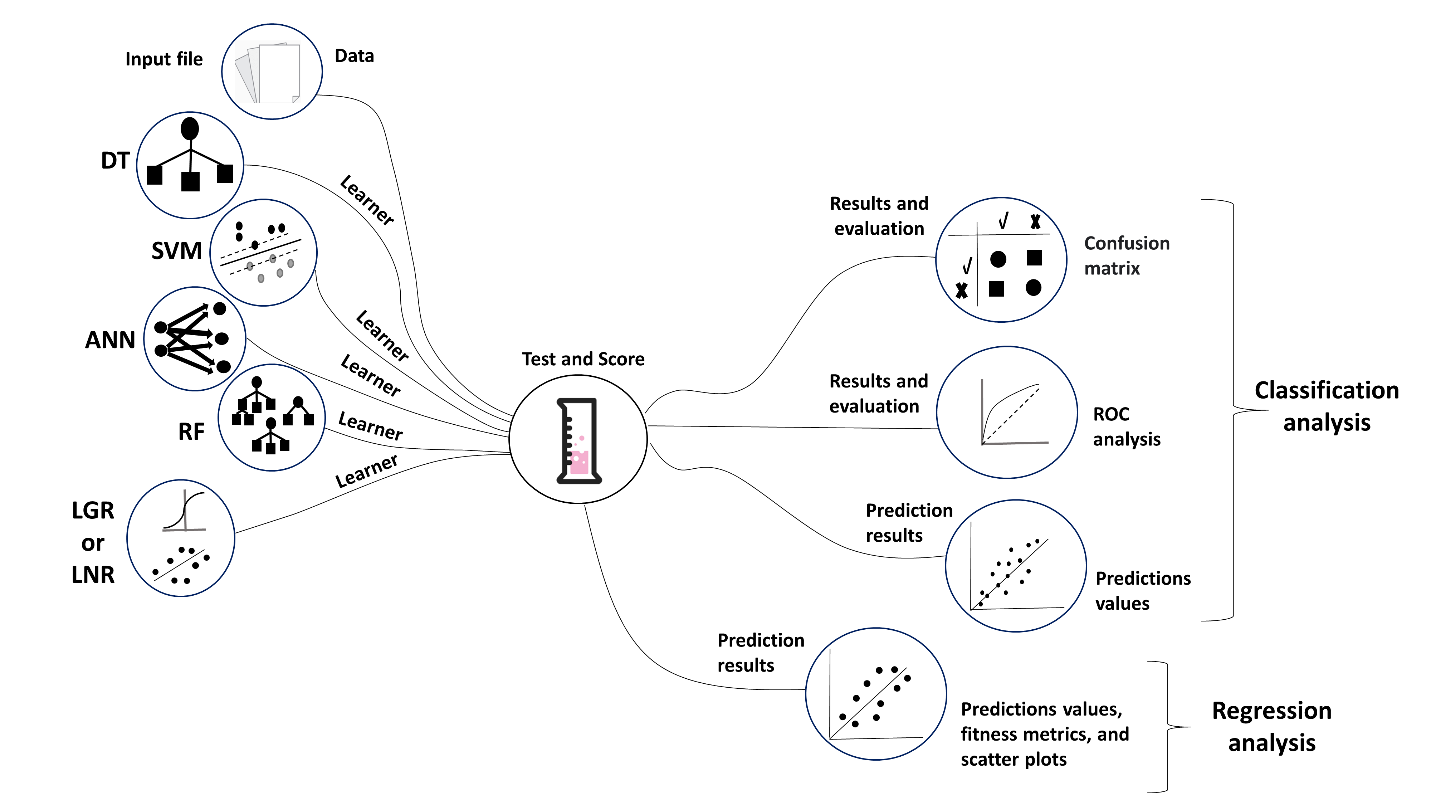


Supplementary Figure S1. Workflow system of Orange software (version 3.24.0). Classification and regression analyses of artificial neural networks (ANN), support-vector machine (SVM), random forest (RF), decision trees (DT), logistic regression (LGR), and linear regression (LNR) machine-learning models. ROC analysis stands for [Receiver Operating Characteristic analysis. LGR was used in classification and LNR in regression analysis.](https://www.statisticshowto.com/receiver-operating-characteristic-roc-curve/)
