# Supplementary material for: Modeling risk of Sclerotinia sclerotiorum-induced disease development on canola and dry bean using machine learning algorithms
Source: Sci Rep. 2022 Jan 17;12:864. doi: 10.1038/s41598-021-04743-1 (PMC8764076; doi:10.1038/s41598-021-04743-1)
Supplement: Supplementary file 2 — Supplementary Table S1. [file 41598_2021_4743_MOESM2_ESM.docx]

**Table S1.** Confusion matrices of five machine learning algorithms including artificial neural network (ANN), random forest (RF), decision tree (DT), logistic regression (LGR), and support-vector machines (SVM) used in classification analyses of canola and dry bean studies. True negative or specificity indicates correct prediction of the healthy plants (incidence ≤20%). False positive is the rate of healthy plants that model predicts them as infected. False negative represents number of cases that were infected but model predicts them as healthy. True positive or sensitivity shows correct prediction of infected plants (incidence>20%).

| Study | Algorithm | True negative | False positive | False negative | True positive | Total |
| --- | --- | --- | --- | --- | --- | --- |
|  |  |  |  |  |  |  |
| Canola | ANN | 38 | 8 | 7 | 78 | 131 |
|  | SVM | 37 | 9 | 7 | 78 | 131 |
|  | RF | 35 | 11 | 8 | 77 | 131 |
|  | DT | 31 | 15 | 14 | 71 | 131 |
|  | LGR | 26 | 20 | 8 | 77 | 131 |
|  |  |  |  |  |  |  |
| Dry bean | ANN | 29 | 3 | 2 | 26 | 60 |
|  | SVM | 28 | 4 | 2 | 26 | 60 |
|  | LGR | 28 | 4 | 3 | 25 | 60 |
|  | RF | 28 | 4 | 5 | 23 | 60 |
|  | DT | 27 | 5 | 5 | 23 | 60 |
|  |  |  |  |  |  |  |
